# Lineage-dependent role of miR-410-3p as oncomiR in gonadotroph and corticotroph pituitary adenomas or tumor suppressor miR in somatotroph adenomas via MAPK, PTEN/AKT, and STAT3 signaling pathways

**DOI:** 10.1007/s12020-019-01960-7

**Published:** 2019-06-04

**Authors:** Tomasz M. Grzywa, Klaudia Klicka, Beata Rak, Dawid Mehlich, Filip Garbicz, Grzegorz Zieliński, Maria Maksymowicz, Emir Sajjad, Paweł K. Włodarski

**Affiliations:** 1 Center for Preclinical Research, The Department of Methodology, Medical University of Warsaw, 1B Banacha Str., 02-097 Warsaw, Poland; 2 Center for Preclinical Research, The Department of Histology and Embryology, Medical University of Warsaw, 1B Banacha Str., 02-097 Warsaw, Poland; 3 Postgraduate School of Molecular Medicine, Warsaw, Poland; 4 The Department of Internal Diseases and Endocrinology, Public Central Teaching Hospital, Medical University of Warsaw, 1A Banacha Str., 02-097 Warsaw, Poland; 5 Laboratory of Experimental Medicine, Centre of New Technologies, University of Warsaw, 2C Banacha Str., 02-097 Warsaw, Poland; 6 Department of Experimental Hematology, Institute of Hematology and Transfusion Medicine, 14 Indiry Gandhi Str., 02-776 Warsaw, Poland; 7 The Department of Neurosurgery, Military Institute of Medicine, 128 Szaserów Str., 04-141 Warsaw, Poland; 8 The Department of Pathology and Laboratory Diagnostics, M. Skłodowska-Curie Memorial Cancer Centre and Institute of Oncology, 5 Roentgena Str., 02-781 Warsaw, Poland

**Keywords:** miR-410-3p, Pituitary adenoma, Proliferation, Invasiveness, MicroRNA, PTEN

## Abstract

**Purpose:**

miR-410-3p plays opposite roles in different cancers and may act as an oncomiR or tumor suppressor miR. The purpose of this study was to assess the role of miR-410-3p in somatotroph, gonadotroph, and corticotroph pituitary adenomas.

**Methods:**

Tissue samples were obtained from 75 patients with pituitary adenoma. miR-410-3p expression was assessed using qRT-PCR performed on RNA isolated from fresh frozen samples. In vitro experiments were performed on cell lines derived from somatotroph (GH3), gonadotroph (RC-4B/C), and corticotroph (AtT-20) pituitary tumors. Cells were transfected with synthetic mimic of miR-410-3p or non-targeting scrambled-miR control. Subsequently, proliferation assays and transwell invasion assays were performed. The expression of cyclin D1, E1, and B1 in cells after transfection was determined using qRT-PCR. The activation of MAPK, PTEN/AKT and STAT3 signaling pathways were assessed using western blot.

**Results:**

We have found that the level of expression of miR-410-3p differs in particular types of pituitary adenomas. miR-410-3p significantly upregulates proliferation and invasiveness of RC-4B/C and AtT-20 cells, while inhibiting GH3 cells. We observed that the levels of cyclin B1 upon transfection with miR-410-3p mimic were increased in RC-4B/C and AtT-20, yet decreased in GH3 cells. We have shown that miR-410-3p promoted the activation of MAPK, PTEN/AKT, and STAT3 signaling pathways in RC-4B/C and AtT-20 cells, but suppressed their activity in GH3 cells.

**Conclusions:**

miR-410-3p acts as an oncomiR in gonadotroph and corticotroph adenoma cells, while as a tumor suppressor miR in somatotroph adenoma cells.

## Introduction

Pituitary adenomas are benign tumors of the sellar region which arise from the anterior lobe of the pituitary gland. They represent the third most frequent intracranial tumors [1].

miRs (microRNAs, miRNAs) are single-stranded, stable, non-coding small RNA molecules, consisting of about 21–24 nucleotides. They play an important role in post-transcriptional regulation of gene expression by suppressing mRNA translation and also reducing mRNA stability. Thus, they control the pathogenesis of the majority of diseases and multiple types of human cancer [2]. miRs regulate all hallmarks of cancer, including the proliferation and invasiveness [3–5]. According to the predominant function of given miR in a cancer cell, we can distinguish oncomiRs and tumor suppressor miRs [6]. Recent studies show a significant impact of miRs on the pathogenesis of pituitary adenomas [7–14].

A number of articles, including our previous studies [15, 16] indicate miR-410-3p may act as a tumor suppressor miR in pancreatic, breast, bone, endometrial, and gastric cancers. However, its function seems to be reversed in some other neoplasms, where it functions as an oncomiR (non-small cell lung, liver, and colorectal cancers). It promotes or inhibits cell proliferation, invasion, migration, and apoptosis [17].

Due to the diverse functions of miR-410-3p in different types of cancer, we decided to examine the role of miR-410-3p in somatotroph, gonadotroph, and corticotroph pituitary adenoma cells.

## Materials and methods

### Patient samples

A retrospective group of 75 patients diagnosed with pituitary adenoma consisting of 34 gonadotroph, 30 somatotroph, 5 corticotroph, 3 plurihormonal, and 3 null cell tumors were enrolled for this study. Tumor samples were retrieved from patients undergoing planned transsphenoidal surgery at the Department of Neurosurgery, Military Institute of Medicine, Warsaw during the years 2014–2016. All tumors were examined by a pathologist. A local ethics committee approved all aspects of this study in accordance with the Helsinki Declaration.

### RNA isolation from tissue, reverse transcription, and quantitative polymerase chain reaction (qPCR)

Total RNA was isolated from 75 fresh frozen tissues: 30 somatotroph, 3 plurihormonal, 34 gonadotroph, 5 corticotroph, and 3 null cell pituitary adenomas using AllPrep DNA/RNA Kit (Qiagen). MicroRNA expression levels were determined using TaqMan Advanced miRNA Assays, hsa-miR-410-3p (assay ID: 001274), hsa-miR-484-5p (assay ID: 478308), and hsa-miR-24 (assay ID: 477992) (Thermo Fisher Scientific) according to the manufacturer's protocol. RNA was reverse transcribed using TaqMan Advanced miRNA cDNA Synthesis Kit (Thermo Fisher Scientific). All qPCRs were performed in MicroAmp Fast Optical 96 Well Reaction Plates (Thermo Fisher Scientific) using Applied Biosystems 7500 Fast Real-Time PCR System with 7500 Software V2.0.6 (Thermo Fisher Scientific). Samples were assayed in triplicates. The obtained Ct values for miR-410-3p as well as mean miR-24 and miR-484 as an endogenous control were used to calculate relative expression using the 2^-∆Ct^ method as described previously [18]. We used the mean Ct values for miR-24 and miR-484 as reference based on our analyses using NormFinder Software [19].

### Cell culture

Pituitary adenoma cell lines: rat plurihormonal with predominantly FSHβ+ and LHβ+ cells [20] RC-4B/C (ATCC® CRL-1903™), therefore referred to as a gonadotroph cell line, rat somatotroph GH3 (ATCC® CCL-82.1™), and murine corticotroph AtT-20 (ATCC® CCL-89™) were obtained from the American Type Culture Collection. Cells were maintained according to the manufacturer's instructions. All cell culture media and reagents were purchased from Gibco BRL (Gran Island, NY, USA).

### Transfection

All transfections were performed using jetPRIME (Polyplus) according to the manufacturer's protocol. miR-410-3p mimic (assay ID: MC11119) and miR-scrambled (miR-scr, miRNA Mimic Negative Control) were obtained from Invitrogen™ mirVana™ (Thermo Fisher Scientific). miRs were used at a final concentration of 50 nM. The efficiency of transfection was confirmed using qRT-PCR (Supplementary Fig. 1)

### Proliferation and colony formation assay

For proliferation assay, 5 × 10^4^ cells/well were seeded in 12-well plates 24 h after transfection and were incubated for 72 h. Then, cells were fixed with 4% PFA and stained with 0.1% crystal violet. For colony formation assay, 2.5 × 10^3^ cells/well were seeded in 6-well plates 24 h after transfection. Cells were incubated 14 days with medium change every 3 days. Then, they were fixed and stained. For both proliferation and colony formation assays, AtT-20 cells were suspended in PBS and photographed. The photos were analyzed using ImageJ (National Institutes of Health, Bethesda MD, USA) and ColonyArea plugin [21].

### MTT

CellTiter 96® Aqueous One Solution Cell Proliferation Assay (Promega) was performed according to the manufacturer's protocol. 5 × 10^3^ cells/well in 96-well plates were seeded 24 h after transfection and incubated for 48, 96, 144, 192, and 240 h. Absorbance at 570 nm was measured using FLUOstar OPTIMA (BMG Labtech).

### Invasion assay

Cells were transfected as described above. VWR® Tissue Culture Plate Inserts, PET Membrane 8.0 µm (VWR) were coated with 100 µl of Matrigel (Corning) in serum-free medium in final concentration 250 µl/ml. 1.5 × 10^5^ cells in 250 µl half-supplemented medium (7.5% FBS for RC-4B/C, 1.25% FBS and 7.5% horse serum for GH3 and AtT-20) were seeded 24 h after transfection in inserts placed in 24-well plates filled with full medium (15% FBS or 2.5% FBS and 15% horse serum, respectively). After overnight incubation, 250 µl serum-free medium was added to inserts. After 24 h, the non-invading cells were removed from the upper surface of the membrane. The invading cells on the lower surface of the membrane were fixed with methanol and stained with 0.1% crystal violet in 20% ethanol. Five photos of each insert were taken with Nikon Eclipse TE2000-U. The photos were analyzed using ImageJ (National Institutes of Health, Bethesda MD, USA).

### RNA isolation from cells, reverse transcription, and qRT-PCR

Total RNA was isolated from cells 24 h after transfection using AllPrep DNA/RNA (Qiagen). Up to 5 µg of RNA was subjected to reverse transcription using GoScript Reverse Transcription Kit (Promega). Real-time qPCR was performed using Power SYBR™ Green PCR Master Mix (Thermo Fisher Scientific). The sequences of primers are shown in Supplementary Table 1. The mean Ct values of the target gene and GAPDH as an endogenous control in miR-410-3p-transfected cells and miR-scrambled as a control were used to calculate relative expression using the 2^-∆∆Ct^ method.

### Western blot

The proteins were extracted from cells 48 h after transfection using Cell Lysis Buffer (cat. no. 9803; Cell Signaling Technology, Inc.), subsequently resolved on 12% polyacrylamide gels, and transferred to PVDF membranes (Millipore, Temecula, CA, USA). Membranes were blocked in 5% bovine serum albumin (Sigma) in TBST buffer and incubated overnight at 4 °C with primary antibodies and subsequently incubated with appropriate HRP–labeled secondary antibodies (Santa Cruz Biotechnology) for 1 h at room temperature. The list of primary antibodies is shown in Supplementary Table 2. The blots were visualized with Western Lighting Ultra ECL (PerkinElmer, Surrey, UK) by ChemiDoc Imaging System (Biorad, Hercules, CA, USA). The level of expression was assessed using densitometric analysis in ImageJ (National Institutes of Health, Bethesda MD, USA). For each western blot β-actin was used as a loading control. Relative expression of proteins in miR-410-3p-transfected cells was calculated in relation to miR-scr-transfected cells. Relative levels of phosphorylated protein forms were additionally calculated in relation to total proteins followed by a normalization to miR-scr-transfected cells.

### Statistical analyses

All experiments were performed in triplicates. Appropriate statistical test, Mann–Whitney test, unpaired *t*-test, paired *t*-test or Kruskal–Wallis test with Dunn’s multiple comparisons test, were applied to assess mean differences between groups. All data were tested using Shapiro–Wilk normality test. All statistical tests were performed using GraphPad Prism 6 (GraphPad Sofware Inc.). All values are represented as mean ± SD or median and interquartile ranges for nonparametric distributions. A *p*-value of <0.05 was considered statistically significant.

## Results

### miR-410-3p is upregulated in invasive tumors compared to noninvasive

**Fig. 1:**
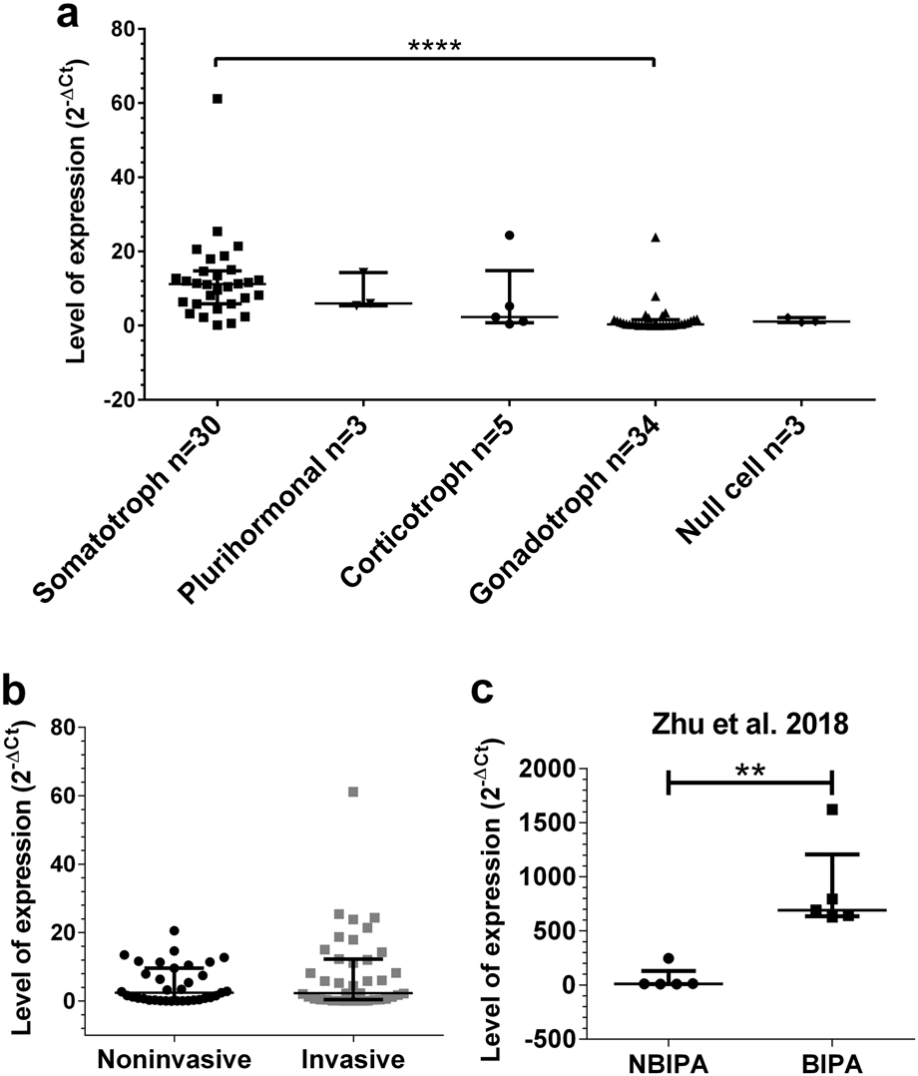
**a** The comparison of the expression of miR-410-3p in different subtypes of pituitary adenomas (*p* < 0.0001, Kruskal–Wallis test). Significantly higher expression in somatotroph tumors compared to gonadotroph (*p* < 0.0001, Dunn’s multiple comparisons test). Median with interquartile range. **b** The comparison of the expression of miR-410-3p in noninvasive (Knosp grade 0 and 1) and invasive (Knosp grade 2, 3, and 4) pituitary adenomas (*p* > 0.05, Mann–Whitney test). Median with interquartile range. **c** Overexpression of miR-410-3p in bone-invasive pituitary adenomas (BIPA) compared to nonbone-invasive pituitary adenomas (NBIPA) (*p* = 0.0031, Mann–Whitney test, data obtained by Zhu et al. [22]). Median with interquartile range. **p < 0.01

We analyzed the level of expression of miR-410-3p in 34 gonadotrophs, 30 somatotrophs, 5 corticotrophs, 3 plurihormonal, and 3 null cell pituitary adenomas (Fig. 1a). The expression level was highest in somatotroph pituitary adenomas (mean 2^-ΔCt^ = 12.19, median = 11.21) while lowest in null cell adenomas (mean = 1.40, median = 1.14) and gonadotroph adenomas (mean = 1.66, median = 0.3592). The mean expression of miR-410-3p in corticotroph tumors was 6.72 (median = 2.29) while in plurihormonal it was 8.54 (median = 5.99). We did not observe a statistically significant difference between the expression of miR-410-3p in invasive tumors (Knosp grade 2, 3, and 4, mean = 8.15 ± 11.76, median = 2.29) compared to noninvasive (Knosp grade 0 and 1, mean = 4.76 ± 5.48, median = 2.40), largely due to a considerable heterogeneity in miR-410-3p expression that we observed (Fig. 1b, *p* > 0.05). However, based on analysis of an expression dataset published by Zhu et al. [22], miR-410-3p was significantly overexpressed in bone-invasive pituitary adenomas (BIPA) compared to nonbone-invasive pituitary adenomas (NBIPA) (fold change 15.13, *p* = 0.0031) Fig. 1c.

### miR-410-3p upregulates proliferation and invasion in RC-4B/C and AtT-20 cells, but downregulates in GH3 cells

**Fig. 2:**
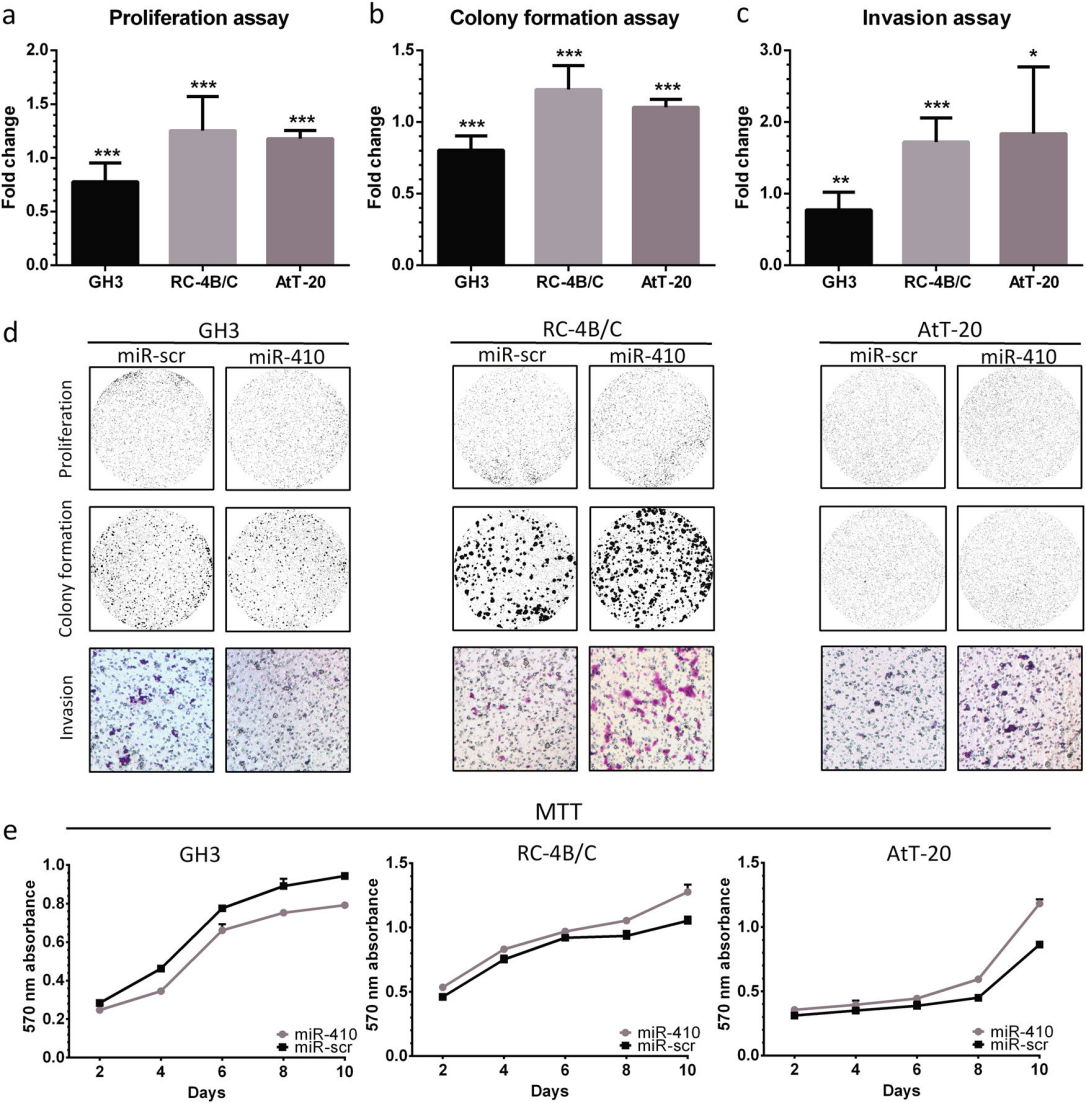
miR-410-3p downregulates GH3 cells proliferation and invasiveness while it upregulates proliferation and invasiveness of RC-4B/C and AtT-20 cells. The results of proliferation assay (**a**), colony formation assay (**b**), and invasion assay (**c**). Mean ± SD. **d** Representative photos of proliferation, colony formation, and invasion assays. **e** The results of MTT. Mann–Whitney test *p < 0.05, **p < 0.01, ***p < 0.001

To identify the effect of miR-410-3p on cells proliferation in pituitary adenoma cells, we performed MTT and proliferation assays using model cell lines. miR-410-3p promoted proliferation in RC-4B/C and AtT-20 cells, while it reduced the proliferative potential of GH3 cells. Based on proliferation assay, miR-410-3p increased RC-4B/C cells proliferation (1.25, *p* = 0.0008) and AtT-20 (1.18, *p* = 0.0002), while decreased GH3 cells proliferation (0.78, (*p* = 0.0008) (Fig. 2a). We found that relative colony formation was higher in miR-410-3p-transfected RC-4B/C cells (1.23, *p* < 0.0001) and AtT-20 cells (1.10, *p* = 0.0003) but lower in miR-410-3p-transfected GH3 cells (0.80, *p* < 0.0001), Fig. 2b.

In order to dissect the role of miR-410-3p in the regulation of pituitary adenomas invasiveness, we performed transwell invasion assays with membranes coated with Matrigel. The results are shown in Fig. 2c. We found that miR-410-3p downregulated invasiveness in GH3 cells with the relative invasion of 0.78 compared to miR-scr (*p* = 0.0094). Conversely, miR-410-3p substantially upregulated cell invasiveness in RC-4B/C cells with the relative invasion 1.72 (*p* < 0.0001) as well as in AtT-20 cells with the relative invasion 1.84 (*p* = 0.0128).

Representative photos of proliferation assay, colony formation assay, and invasion assay are shown in Fig. 2d. Furthermore, MTT assay showed that the differences in proliferation between miR-410-3p-transfected cells and control cells increased with the incubation time (Fig. 2e).

### miR-410-3p-dependent regulation of cell cycle

**Fig. 3:**
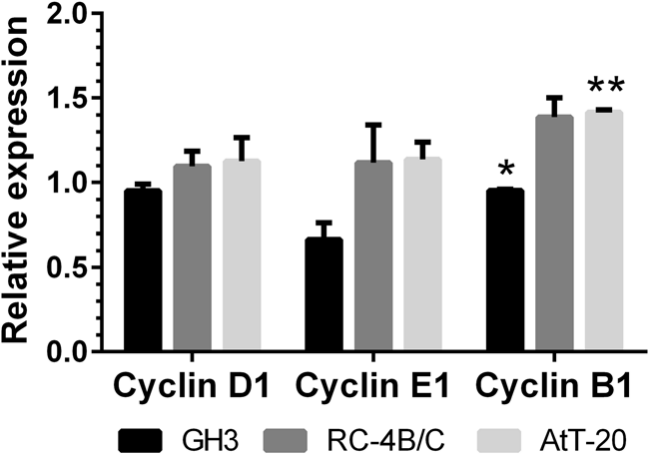
The relative expression of cyclin D1, E1, and B1 on mRNA level in miR-410-3p-transfected cells, normalized to miR-scrambled-transfected cells. Mean ± SD. Paired *t*-test *p < 0.05, **p < 0.01

**Fig. 4:**
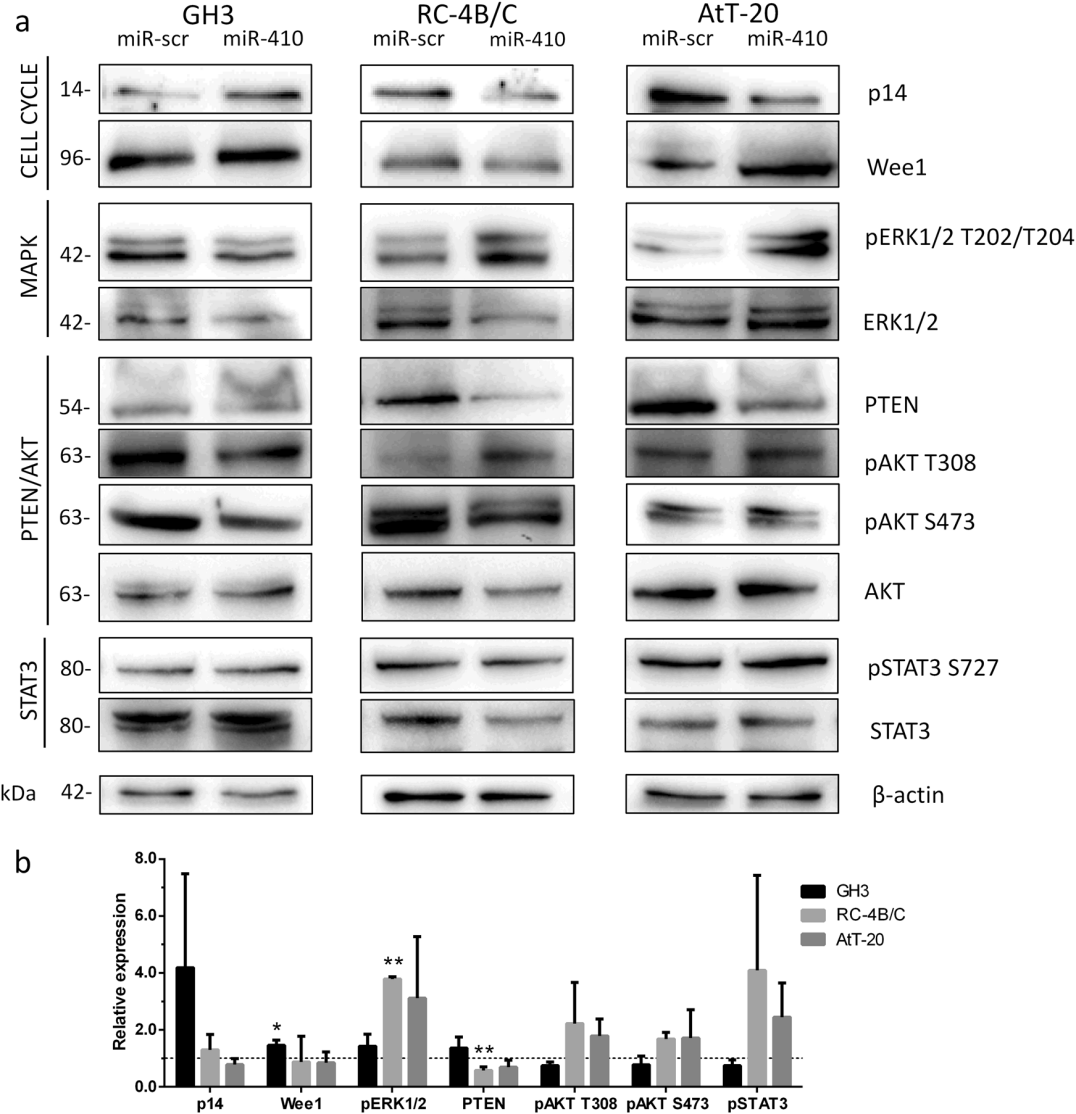
miR-410-3p regulates cell cycle, MAPK, PTEN/AKT and STAT3 signaling pathways. **a** Representative western blots and β-actin as a loading control. **b** The results of densitometric analysis of relative p14 and Wee1 expression, pERK1/2:ERK1/2 total ratio, relative PTEN expression, relative pAKT T308:AKT total ratio, pAKT S473:AKT total ratio, and relative pSTAT3:STAT3 total ratio. Normalized to miR-scrambled-transfected cells. Mean ± SD. Unpaired *t*-test *p < 0.05, **p < 0.01, ***p < 0.001

Due to the possibility of cell cycle regulation by miR-410-3p, we checked the expression of cyclin D1 and cyclin E1 that both drive G1/S phase transition as well as cyclin B1 that drives G2/M phase transition (Fig. 3). While no major changes in cyclins D1 and E1 were detected in studied cell lines, miR-410-3p mimic induced expression of cyclin B1 in AtT-20 cells (relative level 1.42, *p* = 0.001) and suppressed in GH3 cells (relative level 0.95, *p* = 0.0259). Moreover, we checked the expression of negative cell cycle regulators p14 (CDKN2A) and Wee1 on protein level (Fig. 4a). We found that miR-410-3p downregulated the level of p14 and Wee1 in RC-4B/C cells and the level of p14 in AtT-20 cells, but upregulated the level of both inhibitors of cell cycle in GH3 cells

### miR-410-3p regulates MAPK and PTEN/AKT pathways

Constitutive activation of oncogenic kinases like AKT [23] or Erk1/2 [24] is often attributed a major role in promoting tumor invasion and proliferation. Since aberrant activation of MAPK and PTEN/AKT signaling pathways has been associated with pathogenesis of pituitary tumors [25–27] we decided to check whether miR-410-3p overexpression affected activation of these pathways differently in the tested cell lines. The results are shown in Fig. 4. We found that miR-410-3p upregulated the phospho-p44/42 MAPK (Erk1/2) (Thr202/Tyr204):total ERK1/2 ratio in all three cell lines, with the highest level in RC-4B/C cells (3.78 ± 0.05, *p* < 0.001) and the lowest in GH3 cells (1.43 ± 0.24, *p* > 0.05). Moreover, we observed a significant downregulation of the level of total ERK1/2 in RC-4B/C cells (0.38 ± 0.09, *p* = 0.0046, unpaired *t*-test) and a mild downregulation in the other two cell lines (0.77 ± 0.24 in GH3, *p* > 0.05, and 0.93 ± 0.48 in AtT-20, *p* > 0.05, unpaired *t*-test). Further, we checked the level of PTEN as well as PTEN-dependent phospho-AKT T308 and mTORC2-dependent phospho-AKT S473. We found miR-410-3p-dependent downregulation of PTEN in RC-4B/C (0.58 ± 0.07, *p* = 0,0319) and AtT-20 cells (0.70 ± 0.14, *p* > 0.05) leading to a mild phosphorylation increase of AKT at T308 (relative level 1.85 ± 0.49 in RC-4B/C and 1.71 ± 0.39 in AtT-20 cells, *p* > 0.05). The level of PTEN in GH3 cells was slightly upregulated (1.38 ± 0.22, *p* > 0.05). In this setting, miR-410-3p caused a slight decrease in the T308 phosphorylation level of AKT (0.86 ± 0.05, *p* > 0.05) in GH3. We also observed a upregulation of pAKT S473 in RC-4B/C and AtT-20 cells (2.81 ± 0.99 and 1.80 ± 0.55, respectively, *p* > 0.05) and downregulation in GH3 cells (0.83 ± 0.18, *p* > 0.05). The level of pAKT T308/total AKT compared to pAKT S473/total AKT was higher in RC-4B/C cells while lower in AtT-20 cells.

### miR-410-3p-dependent regulation of STAT3

miR-410-3p is also a known modulator of STAT3 (Signal Transducer and Activator of Transcription 3) activity [28]. We found upregulation in the phosphorylation of STAT3 (S727) in both RC-4B/C (4.09 ± 1.93, *p* > 0.05) and AtT-20 cells (2.45 ± 0.69, *p* > 0.05) (Fig. 4). On the other hand, miR-410-3p downregulated this signaling axis in GH3 cells (0.75 ± 0.12, *p* > 0.05). Even though we observed a downregulation of total STAT3 in RC-4B/C cell (0.37 ± 0.19, *p* > 0.05) the cells maintained a net increase in pSTAT3.

## Discussion

In this study, we demonstrate that miR-410-3p plays a lineage-dependent role in pituitary adenomas. There is an increasing evidence that the role of miR-410-3p in molecular oncology is tissue- and context-dependent. miR-410-3p may act as both oncomiR or tumor suppressor miR in different types of cancer [17]. It regulates crucial cellular processes including cell cycle, apoptosis, migration, and invasion. Müssnich et al. found that miR-410-3p is downregulated in gonadotroph adenomas compared with normal pituitary gland [29]. Moreover, they suggested that the downregulation of expression of miR-410-3p is specific to gonadotroph tumors because they noted upregulation of its expression in the majority of adenomas originating from mammosomatotroph cell lineage, i.e., somatotroph and lactotroph tumors. Further, they reported that miR-410-3p targets cyclin B1 and acts as a tumor suppressor miR. However, their conclusions were based on experiments performed only on HEK-293 cells, which does not reflect the unique biology of pituitary tumors. Similarly, Cheunsuchon et al. found downregulation of miR-410-3p expression in nonfunctioning pituitary adenomas, mainly gonadotroph tumors, and its upregulation in GH-, PRL-, and ACTH-secreting tumors [30]. We found that the expression of miR-410-3p was the highest in somatotroph pituitary adenomas, while it was lower in gonadotroph adenomas (*p* < 0.0001, Dunn’s multiple comparisons test). We found that the expression of miR-410-3p was higher in invasive compared to noninvasive tumors, however, it was not statistically significant due to a high heterogeneity in its expression. Zhu et al. [22]. found that the expression of miR-410-3p was higher in bone-invasive pituitary adenoma compared to nonbone-invasive pituitary adenomas. The inconsistency between the expression of miR-410-3p in tissue samples and its role *in vitro* suggests a more complex role of miR-410-3p in pituitary adenomas in vivo, that has to be further investigated.

The results we present in this manuscript indicate that miR-410-3p plays different roles in adenomas originating from different lineages. It acts as oncomiR in RC-4B/C and AtT-20 cells and as tumor suppressor miR in GH3 pituitary adenoma cells. miR-410-3p upregulates pERK1/2 level and downregulates PTEN in RC-4B/C cells as well as upregulates cyclin B1 in AtT-20 cells. However, it upregulates Wee1 and downregulates cyclin B1 in GH3 cells.

Recently, it has been suggested that cyclins and cyclin-dependent kinases are crucial in the development and progression of pituitary adenomas [31–33]. The miR-dependent regulation of cell cycle is important in the biology of pituitary adenoma [9]. Mitosis initiation is controlled by activation of a complex composed of cyclin B and CDK1 (cyclin-dependent kinase 1). In breast cancer and glioblastoma cells, miR-410-3p leads to growth inhibition through targeting CDK1 [34]. The expression of miR-410-3p may be induced by p16^INK4a^, the inhibitor of G1-phase kinases, CDK4 and CDK6 [34]. In the pathogenesis of pituitary adenomas, the regulation of G2/M phase transition and cyclin B1 caught particular attention. Its expression is higher in invasive compared to noninvasive pituitary adenomas [35]. We found that miR-410-3p mildly downregulated the expression of cyclin B1 in GH3 cells and upregulated in AtT-20 cells. We have also investigated the effect of miR-410-3p on negative cell cycle regulators. The Wee1 kinase is a cell cycle inhibitor that interacts with CDK1 and induces G2/M arrest. CDK1 is subject to inhibitory phosphorylation on Y15 catalyzed by Wee1 kinase [36]. Katayama et al. found that it is one of the signaling nodes through which AKT orchestrates cell cycle progression [37]. Wee1 can be phosphorylated by AKT at S642, which induces its nuclear-to-cytoplasmic translocation and progression through the G2/M checkpoint. The expression of Wee1 is decreased in pituitary adenomas and is regulated by miRs [9, 38]. We found that the expression of Wee1 was regulated in a miR-410-3p-dependent manner. miR-410-3p upregulated the level of Wee1 as tumor suppressor miR in GH3 cells. We also observed mildly downregulation of Wee1 by miR-410-3p in RC-4B/C (0.88 ± 0.52) and AtT-20 cells (0.84 ± 0.22).

MAPK and PTEN/AKT are the main signaling pathways responsible for the proliferation and invasiveness of cancer cells and play an essential role in tumor development and progression [25]. Their aberrant activation has been also described as an integral part of pituitary tumorigenesis [25, 26]. Some authors describe synergistic interactions between both RAF/MEK/ERK1/2 and PI3K/AKT signaling pathways to maintain cell homeostasis and its disruption may lead to tumorigenesis [39, 40]. Both pathways are involved in the regulation of cyclin D1 and c-MYC expression. The inhibition of PI3K/AKT/mTOR seems to be a promising therapeutic tool for aggressive somatotroph tumors [41, 42] while ERK1/2 may be a useful therapeutic target to control GH secretion [43].

We found that miR-410-3p upregulated pERK1/2 in all three cell lines, especially in RC-4B/C and AtT-20. The downregulation of total ERK1/2 in cell lines may result from indirect regulation or by unconfirmed direct binding to 3′-UTR as ERK1/2 is a predictive target of miR-410-3p (TargetScan [44]). No data has been published so far concerning the possible mechanism of MAPK regulation by miR-410-3p and this finding warrants further investigation.

PTEN (phosphatase and tensin homologue) is one of the most important tumor suppressors [45]. It directly opposes the activity of PI3K by dephosphorylation of phosphatidylinositol‑3,4,5‑trisphosphate (PIP_3_) leading to the suppression of PDK1/AKT pathway. PTEN inactivation is one of the most common alterations in human cancers [46]. miR-410-3p targets and downregulates the expression of PTEN in prostate cancer leading to activation of AKT/mTOR pathways and promotion of cancer progression [47]. The expression of PTEN is downregulated in pituitary adenomas compared to normal pituitary tissues as well as in invasive adenomas compared to noninvasive [4, 48]. It was shown that miR-17-5p, miR-20a, miR-106b, and miR-200c target PTEN leading to the activation of AKT and increased cell proliferation and invasiveness [48–51]. We found that miR-410-3p downregulated PTEN as well as it upregulated pAKT T308 and pAKT S473 in RC-4B/C and AtT-20 cells. However, miR-410-3p in GH3 cell line mildly upregulated PTEN which led to the downregulation of AKT phosphorylation. This could be one of the reasons for the observed differences in cell proliferation and invasion between these cell lines.

Next, we checked the effect of miR-410-3p on STAT3, a critical regulator of malignant transformation and tumor progression [52]. MicroRNAs are emerging as important regulators of STAT3 in the cancer pathogenesis [53]. In endothelial cells [54] and CD3+ T cells [28] miR-410-3p directly targets STAT3 and downregulates its expression. We found that miR-410-3p downregulated the level of STAT3 only in RC-4B/C cells (relative expression 0.37 ± 0.19). However, miR-410-3p upregulated the net level of phospho-STAT3 in both RC-4B/C and AtT-20 cells, while it was downregulated in GH3 cells (Fig. 4). It may be due to the downregulation of SOCS3 (suppressors of cytokine signaling 3), an inhibitor of JAK kinase activity and STAT phosphorylation, by miR-410-3p [55]. Interestingly, the S727 phosphorylation site we studied is directly regulated by MAPK [56] and mTOR [57] kinases. Since the changes in S727 phosphorylation that we observe mirror the changes in pERK1/2, MAPK activity could be one of the factors contributing to the changes in STAT3 activity that we report. STAT3 is a transcription factor involved in cell proliferation, transformation, and invasion [58]. It was shown that STAT3 induced GH hypersecretion in somatotroph tumors [59]. However, there is no correlation between STAT3 expression and pituitary tumor invasiveness [60].

## Conclusions

We have shown that miR-410-3p plays an opposite role as an oncomiR in gonadotroph RC-4B/C and corticotroph AtT-20 pituitary adenoma cells while as tumor suppressor miR in somatotroph GH3 pituitary adenoma cells. It regulates tumor cells proliferation and invasiveness through MAPK, PTEN/AKT, and STAT3 signaling pathways. We have shown that single miR may act differentially in different types of pituitary adenomas.

## Supplementary information

Supplementary information
