# Supplementary material for: Lineage-dependent role of miR-410-3p as oncomiR in gonadotroph and corticotroph pituitary adenomas or tumor suppressor miR in somatotroph adenomas via MAPK, PTEN/AKT, and STAT3 signaling pathways
Source: Endocrine. 2019 Jun 4;65(3):646–55. doi: 10.1007/s12020-019-01960-7 (PMC6717603; doi:10.1007/s12020-019-01960-7)
Supplement: Supplementary file 1 — Supplementary information [file 12020_2019_1960_MOESM1_ESM.docx]

**Supplementary figure 1.** The efficiency of miR-410-3p transfection. The obtained CT values for miR-410-3p and mean for miR-24 and miR-484 as an endogenous control for miR-410-3p-transfected and miR-scrambled-mimic were used to calculate relative expression using the 2^-^^∆∆Ct^ method. RNA was isolated 24h after transfection.

Supplementary table 1.

| Target | Forward | Reverse |
| --- | --- | --- |
| Cyclin D1, rat | 5`-TGCTTGGGAAGTTGTGTTGG-3` | 5`-AATGCCATCACGGTCCCTAC-3 |
| Cyclin D1, mouse | 5`-TCCCAGACGTTCAGAACC-3` | 5`-AGGGCATCTGTAAATACACT-3` |
| Cyclin E1, rat | 5`-AGAGCAGCAGGGGTCTACAA-3` | 5’-CCATGGCCTCCTTAACTTCA-3` |
| Cyclin E1, mouse | 5`- TGCACCAGTTTGCTTATGTT-3` | 5`-CCGTGTCGTTGACATAGG-3` |
| Cyclin B1, rat | 5`-AAAGGCGTAACTCGAATGGA-3` | 5`-CCGACCTTTTATTGAAGAGCA-3` |
| Cyclin B1, mouse | 5`-AGCAAATATGAGGAGATGTACC-3’ | 5’-CGACTTTAGATGCTCTACGGA-3` |
| GAPDH, rat | 5`-TGGGAAGCTGGTCATCAAC-3` | 5`-GCATCACCCCATTTGATGTT-3’ |
| GAPDH, mouse | 5`-GCAGTGGCAAAGTGGAGATT-3` | 5`-GAATTTGCCGTGAGTGGAGT-3` |

Supplementary table 2.

| Target | Dilution | Cat. no. | Source |
| --- | --- | --- | --- |
| phospho-p44/42 MAPK (Erk1/2) (Thr202/Tyr204) | 1:1000 | 4370 | Cell Signaling Technology, Inc. |
| total-p44/42 MAPK (Erk1/2) | 1:1000 | L34F12 | Cell Signaling Technology, Inc. |
| PTEN | 1:500 | 9188 | Cell Signaling Technology, Inc. |
| phospho-AKT (Ser473) | 1:1000 | 4060 | Cell Signaling Technology, Inc. |
| phospho-AKT (Thr308) | 1:1000 | C31E5E | Cell Signaling Technology, Inc. |
| total-AKT | 1:1000 | 11E7 | Cell Signaling Technology, Inc. |
| CDKN2A/p14ARF | 1:500 | GTX129902 | GeneTex |
| Wee1 | 1:200 | sc-5285 | Santa Cruz Biotechnology |
| phospho-STAT3 (S727) | 1:500 | sc-8001-R | Santa Cruz Biotechnology |
| total STAT3 | 1:1000 | ab705 | Abcam |
| β-actin | 1:500 | Sc-47778 | Santa Cruz Biotechnology |
